# Docking-Informed Machine Learning for Kinome-wide Affinity Prediction

**DOI:** 10.1021/acs.jcim.4c01260

**Published:** 2024-12-10

**Authors:** Jordy Schifferstein, Andrius Bernatavicius, Antonius P.A. Janssen

**Affiliations:** 1 Department of Molecular Physiology, Leiden Institute of Chemistry, Leiden University, Leiden 2333CC, The Netherlands; 2 Oncode Institute, Utrecht 3521AL, The Netherlands; 3 Leiden Institute of Advanced Computer Science, Leiden University, Leiden 2333CC, The Netherlands

## Abstract

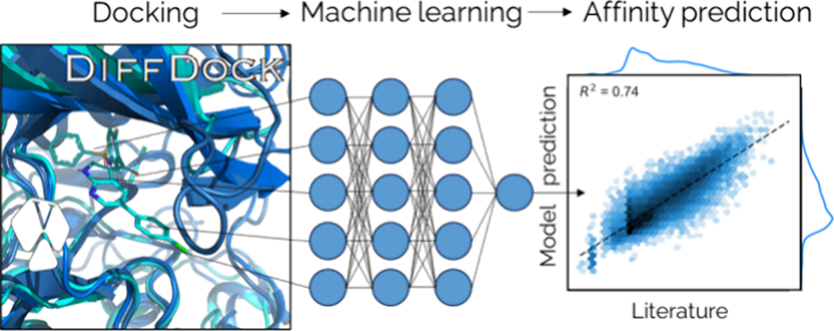

Kinase inhibitors are an important class of anticancer drugs, with 80 inhibitors clinically approved and >100 in active clinical testing. Most bind competitively in the ATP-binding site, leading to challenges with selectivity for a specific kinase, resulting in risks for toxicity and general off-target effects. Assessing the binding of an inhibitor for the entire kinome is experimentally possible but expensive. A reliable and interpretable computational prediction of kinase selectivity would greatly benefit the inhibitor discovery and optimization process. Here, we use machine learning on docked poses to address this need. To this end, we aggregated all known inhibitor-kinase affinities and generated the complete accompanying 3D interactome by docking all inhibitors to the respective high-quality X-ray structures. We then used this resource to train a neural network as a kinase-specific scoring function, which achieved an overall performance (*R*^2^) of 0.63–0.74 on unseen inhibitors across the kinome. The entire pipeline from molecule to 3D-based affinity prediction has been fully automated and wrapped in a freely available package. This has a graphical user interface that is tightly integrated with PyMOL to allow immediate adoption in the medicinal chemistry practice.

## Introduction

Protein kinases are one of the main protein families targeted by anticancer drugs, with 80 approved drugs and around 150 in clinical testing.^1^ However, current FDA-approved kinase inhibitors are designed to target only a few percent of the entire protein family.^2^ The so-far untargeted kinases, thus, offer great opportunities for the development of novel molecular therapies.

The chances of success for any drug greatly depend on two parameters: affinity of the drug for the intended target protein, and selectivity over the rest of the protein family. Off-target activity is often the main cause of (pre)clinical toxicity, and side-effects in general.^3^ This issue is particularly pressing for kinase inhibitors, as these in most cases target the ATP-binding site of the protein, which is highly conserved across this large protein family.^4^ This leads to many kinase inhibitors potently binding to many family members, sometimes inhibiting as much as 70% of all kinases.^5^ Determining the specificity of an inhibitor over all ±500 kinases is experimentally feasible, but is prohibitively expensive in terms of time, material and funds to perform on a routine basis.

In recent years, various computational methods of predicting kinase inhibitor selectivity have thus been developed.^6−8^ Approaches vary from “classical” protein structure-based techniques such as molecular docking to machine learning approaches such as Quantitative Structure Activity Relationship (QSAR) studies. The revolution of artificial intelligence (AI) has not gone unnoticed in this field, and e.g. AlphaFold^9^ will have a tremendous impact in the coming years, giving direct access to structures for all proteins. Structure-based methods typically rely on either classical physics-based scoring functions to “score” a generated protein–ligand complex. More recently, machine learning-based scoring functions such as RFScore have reached state-of-the-art performance.^10−12^ These scoring functions were trained on experimental data sets such as the PDBbind, offering a relatively broad set of protein-inhibitor complexes and their bioactivity data.^13^ Some kinase specific tools have also appeared in recent years. KinomeX, a multitask classification DNN trained only on ligand molecular fingerprints can be classified as one of the QSAR based models.^14^ It was available online as a service but did not provide the option of installing locally for proprietary data applications. KinomeX has more or less been superseded by KinomeMETA of the same research group.^15^ KinomeMETA uses a GNN architecture on ligand structures to predict the Boolean activity on >600 kinases. KinomeMETA is available as an online service, but no local variant is provided. Neither of these models has an obvious way to inspect the prediction origin. Closer to what we envision is ProfKin, a structure-based tool that compares docked poses to 4219 experimentally determined kinase-ligand complexes.^16^ The interaction fingerprint and similarity scoring is used to provide expected kinase targets for a given ligand. This tool was also only available as a server.

We set out to develop a fully automated docking-based tool akin to ProfKin, but with the aim of predicting binding affinities for kinases. As it is generally accepted that pose finding for most docking algorithms is very good,^17^ we envisioned that a large docking-based protein-inhibitor data set for which biochemical data is known should also function as a basis for training a scoring function. We demonstrated this approach by generating protein-inhibitor complexes for all kinase inhibitors in the Papyrus data set,^18^ a large aggregation of literature binding data, for kinases of which a high-quality experimental protein structure is available in the KLIFS database, a kinase specific mirror of the PDB.^19,20^ We used two docking algorithms: Autodock VinaGPU^21^ and DiffDock.^22^ This generated database is then used to train a multilayered Neural Network as scoring function, that shows excellent performance on bioactivity predictions for unseen inhibitors. The automated workflow has been wrapped in an easily installable Docker container^23^ with a convenient PyMOL Graphical User Interface (GUI) plugin, allowing broad access to the methodology.

## Results

### Extracting Literature Biochemical and Structural Data

To generate our desired docking-based training data set, we first needed to select all kinases of which we have a high-quality experimental structure. As a source of well curated and annotated kinase protein structures available in the Protein Data Bank we used the KLIFS database. These structures were filtered based on their resolution (≤2.5 Å) and KLIFS quality metric (≥8). We selected the best of each of the four possible combinations of DFG-in/out and α-C helix in/out as annotated in the KLIFS database. In total, this led to 345 protein structures for 226 unique kinases.

Next, we extracted all reported inhibitory activities for these kinases in the Leiden Papyrus data set, a curated resource combining data from resources such as ChEMBL, PubChem and others. We chose to indiscriminately use pIC_50_, p*K*_i_ and p*K*_d_ values, collectively from hereon: pChEMBL values. We filtered the compounds to entail only the more drug-like small molecules using quite lax criteria (MW ≤ 750, NumHBD ≤ 10, NumHBA ≤ 15, Rotatable Bonds ≤15), which should have reasonable chance to dock well and form a representative training set for real world medicinal chemistry applications. An overview of the resulting physicochemical properties and chemical diversity is plotted in Figure S1.

**Figure 1:**
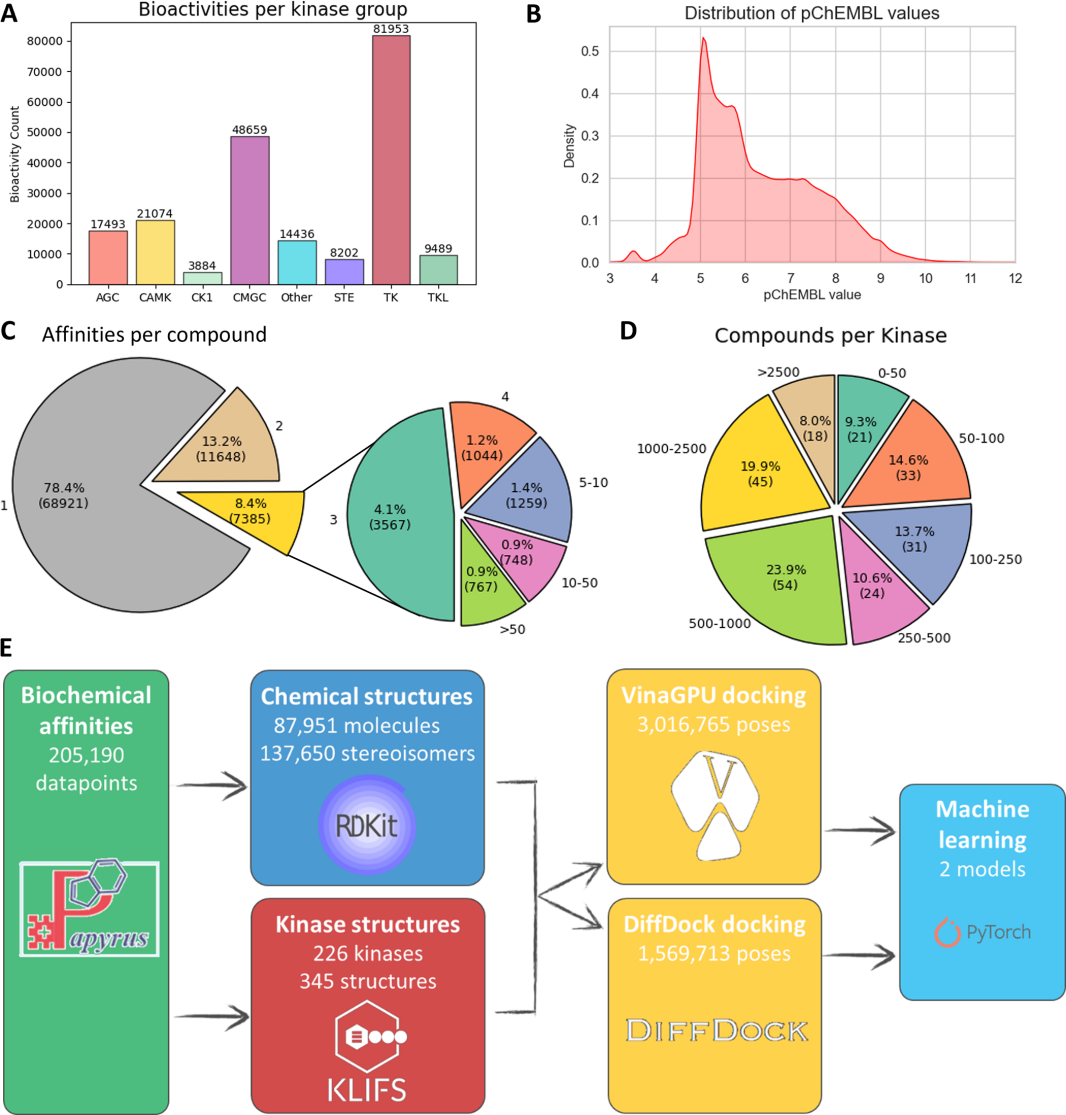
**Kinase activity data set** | A) Distribution of kinase inhibition values reported per kinase group; B) Distribution of the reported inhibitory values; C) Number of pChEMBL values for unique kinases reported per kinase inhibitor, *i.e*., against how many kinases was a compound tested; D) Number of reported inhibitors per kinase, *i.e*., how many compounds were tested for a kinase; E) Overview of the workflow of the work in this paper.

This procedure led to a completed data set of in total 205,190 affinity values for 87,951 unique compounds against 226 unique kinases. A summative view of the workflow and complete resulting data set is depicted in Figure 1 and Figure S1.

### Large Scale Molecular Docking Using Open Source Software

We set up an automated docking pipeline to generate a set of docking poses for all inhibitor-protein pairs in the created data set (Figure 1E). To this end, inhibitors were prepared for docking using an RDKit^24^ pipeline, which enumerates potential stereo- and double bond isomers, and generates a 3D conformer. For each protein structure, a binding site was defined using PyVOL to guide the VinaGPU docking algorithm.^25^ All prepared isomers were consecutively docked in the known targets of these inhibitors using two docking algorithms: Autodock VinaGPU and the diffusion-based DiffDock algorithm (version of December 2022).

For all compound-protein structure pairs, a maximum of 5 poses were generated. The poses were filtered for excessive atomic overlap based on a tailored clash-score (see Methods and Figure S2) to get rid of unphysical poses generated, a problem especially prevalent in DiffDock generated poses. For the inhibitor-kinase pairs in our data set for which an experimental pose has been determined (only ±0.2% of the 205,000), the root mean squared deviation (RMSD) was calculated for both docking algorithms. Median ± absolute deviation for DiffDock and VinaGPU were 1.296 ± 0.587 Å and 5.659 ± 4.177 Å, respectively.

The results of this large-scale docking project were aggregated and have been made available in an SQLite database that holds all activities, compounds, isomers, protein information, kinase structure information and all poses for both docking tools. A simplified schema of this database with statistics per table is depicted in Figure S3A. The database includes all.mol-formatted poses in a compressed format, as well as the.pdb files for all KLIFS structures used. The database was designed to be readily usable for machine learning applications. Additionally, a KNIME-based user interface has been built to browse and query the generated docking complexes (Figure S3B). The full database and accompanying application are freely available on Zenodo and GitHub (*vide infra*).

### Baseline Performance of Readily Available Docking Scores

**Figure 2:**
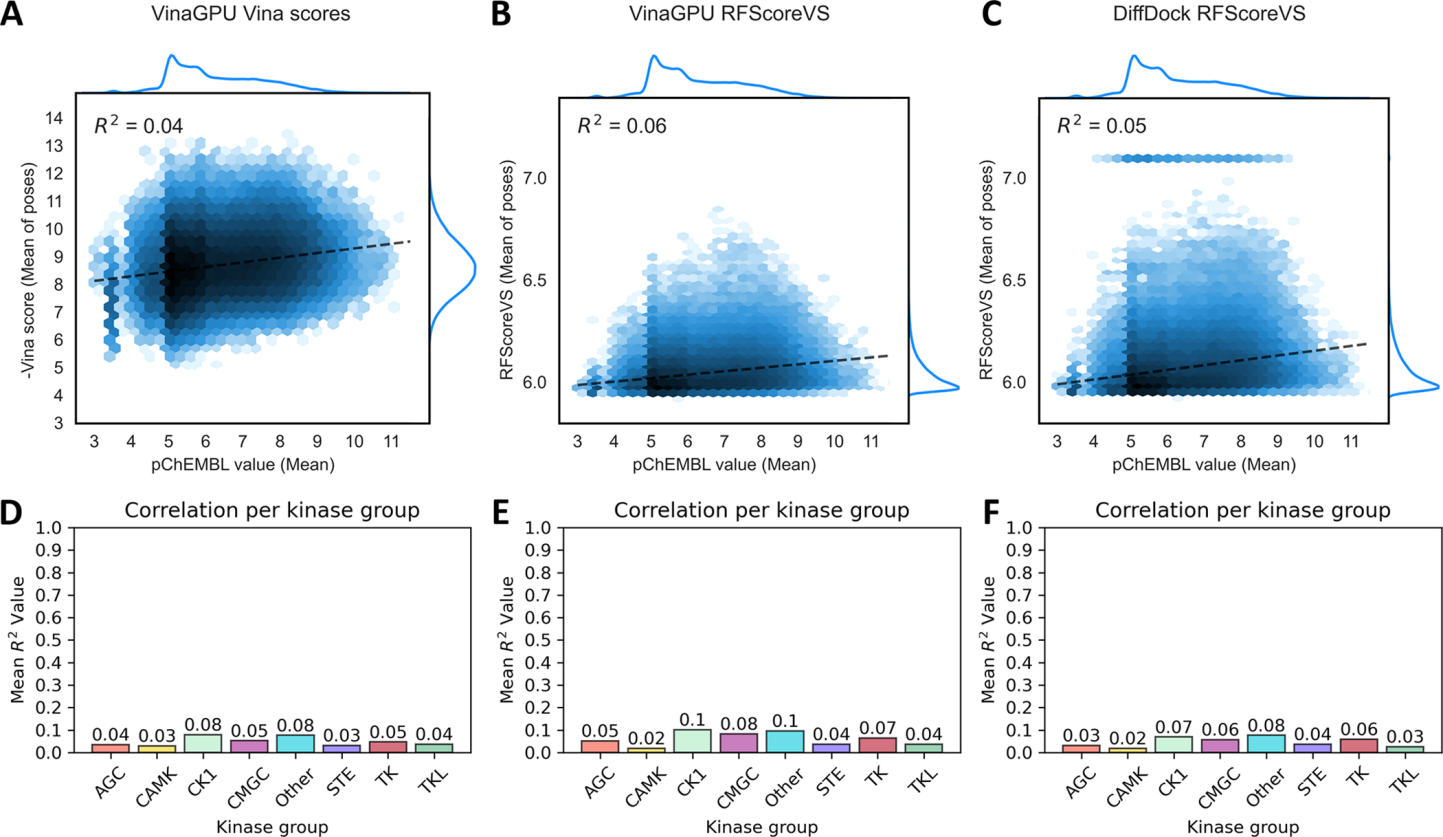
**Correlation of Vina and RFScoreVS scoring functions with Papyrus pChEMBL data** | Predicted affinity values vs literature values displayed as logarithmic hexbin plots, as based on the -Vina score for all VinaGPU poses (A), RFScoreVS for all VinaGPU poses (B), RFScoreVS for all DiffDock poses (C), *R*^2^ calculated per kinase and aggregated per kinase group for Vina scores (D), RFScoreVS for VinaGPU poses (E) and RFScoreVS for DiffDock poses (F).

The performance of two readily available docking scores was assessed to establish a baseline for bioactivity prediction. To this end, we assessed the predicted binding affinity by the Vina score, and used RFScoreVS^26^ to rescore all poses generated by VinaGPU and DiffDock. The results are aggregated in Figure 2. Unsurprisingly, neither of the scoring algorithms showed any productive correlation with the Papyrus pChEMBL values, either when looking at the entire data set (Figure 2A-C) or when aggregating the per-kinase correlation coefficient (*R*^2^) over the kinase groups (Figure 2D-F).

### Kinome-wide Activity Predictions Learned from Docked Poses

**Figure 3:**
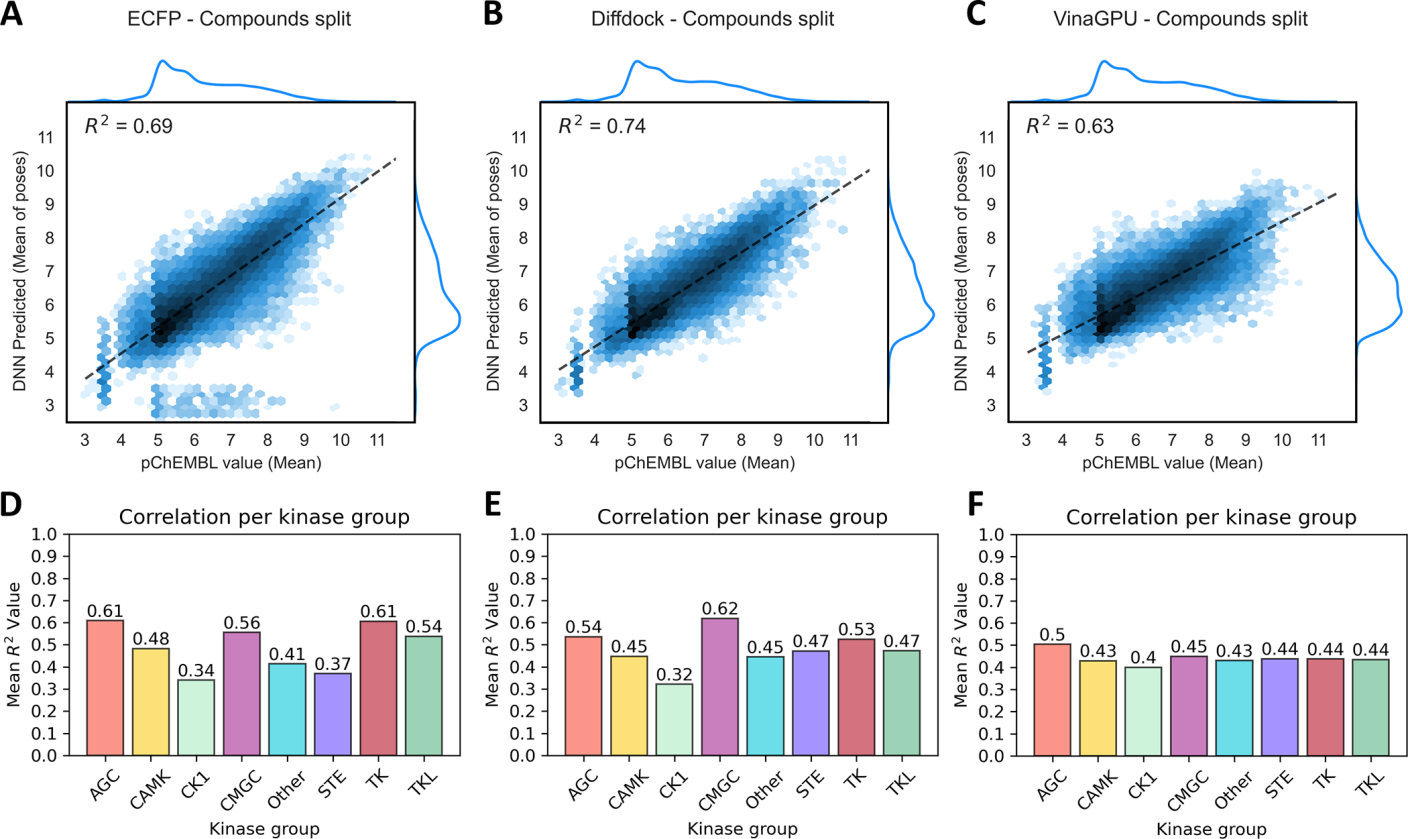
**Model performance** | Predicted affinity values vs literature values for the compounds-split test set displayed as logarithmic hexbin plots, as based on predictions for ECFP models (A), the DNN trained on DiffDock poses (B) and on the VinaGPU poses (C). Panels D, E and F show the average performance per kinase group for ECFP, DiffDock and VinaGPU models, respectively.

We then set out to train a more performant kinase specific scoring function on this unprecedently large structural data set. First, the database was used to generate protein–ligand extended connectivity (PLEC)^27^ fingerprints for the first three poses of every protein structure-inhibitor pair. All PLEC fingerprints were used as input for one single 3-layer Deep Neural Network tasked with predicting the affinity value based on a given fingerprint. This was done separately for the two docking algorithms, to compare their relative performance in this task. The generated models, which function as kinase-specific scoring functions, were trained on either a random 80:20 split of protein-inhibitor activity pairs, an 80:20 compound-based split (completely unseen compounds) or an 80:20 split based on kinases (completely unseen kinases as test set). These latter splits are intended to assess the generalization capabilities of the models toward newly designed inhibitors or unseen kinase targets, respectively. As a nondocking 2D comparison, in parallel we trained the same DNN on only the ECFP4 fingerprints of the inhibitors, to assess the added value of using docked poses as input. In this case we trained one model per kinase for all kinases that had at least 100 unique inhibitors known (172 out of 226 kinases in the data set). The performance results of these models are shown in Figure 3, Figure S4 and Figure S5 and specified per kinase in Supplementary Tables 1–3 (Supporting Information).

Regardless of the underlying docking algorithm, the performance of the DNNs trained on the compound splits (*R*^2^ = 0.63–0.74) vastly outperformed both the original Vina scoring (*R*^2^ = 0.04) as well as the rescoring using RFScoreVS (*R*^2^ = 0.05–0.06). For the DiffDock model, for 86 out of 214 kinases (40%) the *R*^2^ of the compound split was higher than 0.6, yielding predictions of sufficient quality to be genuinely informative in drug discovery projects. This value is comparable to the ECFP models, where 84 out of the 172 were ≥0.6. Of note, the ECFP models were only trained for kinases with ≥100 compounds, which leads to fewer kinases covered overall. The DiffDock model can extrapolate to some extend to the lower coverage kinases that are lacking in de ECFP models, with an *R*^2^ ≥ 0.6 for 18% of these (8 out of 42). The VinaGPU model shows somewhat lower overall performance, with 65 out of all 220 models having an *R*^2^ ≥ 0.6, and 5 of the low-coverage kinases. This corresponds to the overall higher RMSD as observed in the docking procedure, pointing to the lower quality of the underlying training data.

Comparing the DiffDock and VinaGPU-based models shows some intriguing results. There is only a low correlation between the performances per kinase (Figure S6–7). This can partially be attributed to the smaller number of successful docking poses DiffDock generated but could also be due to the intrinsic differences between the pose finding tools.

The different splits clearly showed that the random splits performed best overall, although only slightly outcompeting the compound split. This is to be expected as for 78% of the compounds there is only 1 activity in the data set, meaning that the random and compound splits have highly similar difficulties in practice. However, for unseen kinases the performance drops significantly (Figure S5). This seems to indicate that the model strongly relies on the kinase structure underlying the complexes and suggests that when appending new kinases or KLIFS structures to the data set, retraining of the model is warranted.

### KinaseDocker^2^ Release for Direct Local Application

**Figure 4:**
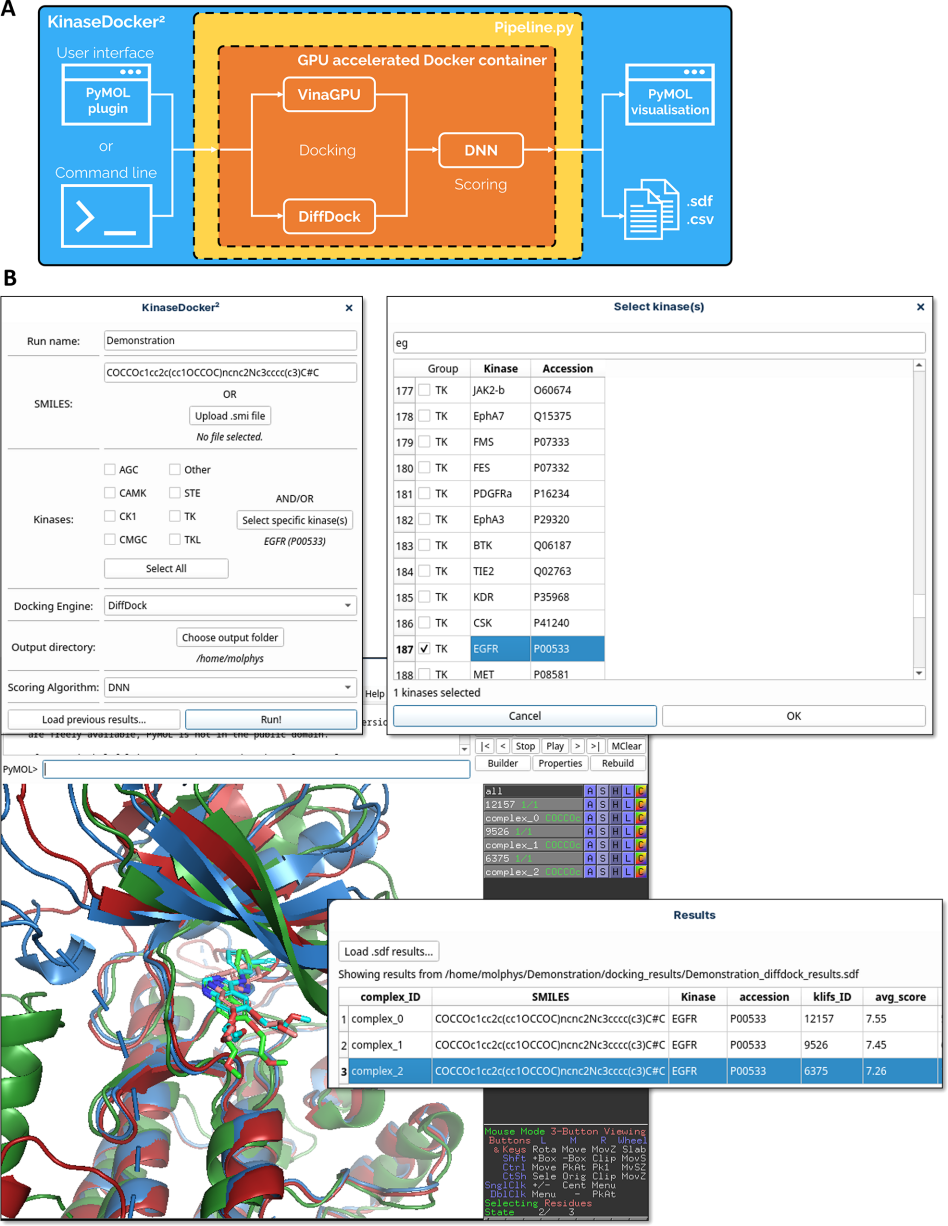
**A user-friendly application: KinaseDocker**^**2**^ | (A) Schematic overview of the software setup; (B) screenshots of the Graphical User Interface of KinaseDocker^2^ showing the initial configuration and kinase selection screens, as well as the result after running the program. The prediction results are shown in a table on screen, with the affinity value (avg_score) and details of the docking run. Docked complexes can be loaded in 3D for further assessment.

Encouraged by the strong performance across the kinome we decided to wrap our workflow and models in a user-friendly application that allows predictions to be generated by a medicinal chemist in real-world applications. Because the model inherently generates docking poses on which the affinity prediction is based, interpretation of the reliability of the output can be done on a per-compound and per-kinase basis. With this interpretability end point in mind, we chose the open-source molecular viewer PyMOL as the basis for the program. Schematically shown in Figure 4A, we built a PyMOL plugin that launches a pipeline to handle the predictions. The docking and consecutive bioactivity prediction by the neural network is handled by a Docker image that requires minimal installation by the user.

The user interface is shown in screenshots in Figure 4B, where the plugin allows the input of a (list of) SMILES strings, the selection of a (list of) kinases and the choice of docking engine. After the prediction is run, the output data is written to files as well as presented in a Results table on screen, showing the generated complexes with their predicted pIC_50_ (avg_score in the screenshot). Generated complexes can be loaded and inspected in the PyMOL session. Programmatic access is available if larger scale runs are desired. The whole codebase has been designed to be modular, allowing the future implementation of different model architectures or structure encodings. All code and Docker images are openly available, see section Code Availability.

## Discussion

The homogeneity of the sources of biochemical data in the Papyrus data set (and nearly every other publicly available data set) inherently means that there is considerable noise in the data. Realistically, *R*^2^ values of around 0.8 are as high as can be achieved when taking experimental error into account.^28^ This means that the DiffDock model for 42 kinases (±20%) already reached this maximum. For these, no significant improvement on this metric can be expected regardless of the methodological improvements or addition of further data. Adding more (diverse) compounds would for these kinases merely expand the chemical space where the model is applicable. For the kinases with lower performing predictions, the addition of more data and/or more structures could still increase performance.

Training (machine learning-based) scoring functions on structural data has been a successful strategy for years, enabled by data sets such as the PDBbind, as demonstrated by, for example, the RFScore series.^12,26,29−31^ Utilizing the accuracy of pose finding in docking algorithms to synthesize an orders of magnitude larger training data set has, to the best of our knowledge, not been attempted before. Here we clearly showed that the approach in the basis works and outperforms current state-of-the-art in this kinase-specific use-case. There are many possibilities for future improvement over the current machine learning implementation. The docking performance of our VinaGPU workflow was not very high, with an average RMSD > 5 Å. More manual curation of the data set could reduce the amount of flawed docking poses, arguably positively impacting the quality of the training data.

From a machine learning perspective, the current choice of encoding the poses (3D) using PLEC fingerprints (1D) and utilizing a basic DNN architecture is inherently lossy. Implementing geometric deep learning models directly on the 3D data could positively impact performance if it can make better use of the available information. Additionally, the attention mechanism of the Transformer architecture could be used to highlight important regions in the complex for the generated prediction, yielding better interpretability and guidance for compound optimization.

There are more domain-focused improvements that could improve the performance too. The current implementation uses every KLIFS structure available for a certain kinase when docking a compound, regardless of inhibitor type (type I, II, III).^32,33^ Previous work has shown that ML models can differentiate Type I and II inhibitors based on structure to a reasonable extend.^34^ By only considering the poses of a molecule in their preferred activity state (DFG-in or -out), when available, the predictions should theoretically be improved. Another limitation is the domain of covalent kinase inhibitors. Though reliable poses can be obtained with known covalent drugs, noncovalent docking poses can never capture the influence of covalent bond formation.

To broaden the scope of kinases for which predictions can be made, structural data on the proteins is currently the main bottleneck. Of the 636 kinases, 226 (±35%) have crystal structures that meet our criteria. Of these, only about 26% (59) have both DFG-in and -out(-like) structures available. A strategy to enrich this data set could be through homology modeling. Considering the high sequence and structural similarity in the kinase domains, for many if not most kinases a reliable homology model in both DFG-states should be feasible to obtain. Adding these to the data set would not only considerably extend the applicability of the model to the entire kinome, it would also grow the size of the available biochemical training data with >100,000 data points for which currently no high quality experimental structure is available.

## Conclusions

Kinase inhibitors are an essential part of anticancer therapy. Developing new kinase inhibitors suitable for clinical use requires these to be as specific as possible, targeting primarily the intended kinase. Due to the high homology in kinase domains, this is not a trivial requirement. Computational tools to aid in the development of these inhibitors by predicting inhibition across the kinome can be of great value. Current state-of-the-art struggles to perform well across the protein family, in part due to the lack of suitable data. Here, we generate a large data set of predicted binding poses, each corresponding to an experimental binding affinity in the Papyrus data set, where a high-quality kinase domain structure of the target is available in the KLIFS database. We showed that this data set forms a strong basis on which to train machine learning models that can predict binding affinities of compounds for a wide variety of targets. We trained a Deep Neural Network on a 1D protein–ligand interaction fingerprint representation (PLEC) and showed that this vastly outperforms readily available (re)scoring functions like Vina score and RFscoreVS. Encouraged by these results, we developed a user-friendly interface to bring the automated docking procedure and scoring function as a freely available tool called KinaseDocker^2^ to the bench chemist. Simultaneously, we ensured the modularity of the code, so that exchanging the protein–ligand complex encoding or the predictive model for more advanced approaches is feasible. Finally, we setup an interface for the database of docking poses to expose the data encapsulated in this to the general (bio)chemist.

We expect that the scoring functions trained here are useful as is, but also that, together with the data set generated here, they form a starting point to further tackle the kinase selectivity question, enabling the reliable prediction of affinities across the kinome to aid in bringing new and safe anticancer drugs to patients.

## Methods

### Biochemical Data

Data was retrieved from Papyrus v5.5,^18^ filtering on the Uniprot Protein Class “Kinase” and data quality “High”. The data was matched to the KLIFS^20^ data set based on Uniprot^35^ accessions. Mutations were disregarded and averages for unique compound – Uniprot pairs were used as activity value (pChEMBL). Included bioactivities were filtered based on the drug-likeness of the measured compounds. Filters used were MW between 250 and 750 Da, rotatable bonds ≤15, number of hydrogen bond donors ≤10 and number of hydrogen bond acceptors ≤15, calculated using RDKit.^24^

### Structural Data

Kinase structures and annotations were retrieved from KLIFS in October 2022. The structures were filtered on resolution (≤2.5 Å) and missing residues (≤5) after which the highest quality (KLIFS metric) structure was selected based on DFG-in/out and αC-helix states as annotated in KLIFS, if available. The.mol2 files were downloaded and converted to PDB files using OpenBabel.^36^ PDB structures thus generated were used as is for DiffDock or further converted to.pdbqt format using the Open Drug Discovery Toolkit^37^ for use with Autodock VinaGPU.

### Docking Benchmark Set

Ligands from the KLIFS database were extracted and used as a benchmark data set for the two docking algorithms used. RMSD was determined using the CalcLigRMSD extension for RDkit.^38^

### Pocket Definition

Pockets for Autodock Vina docking were automatically generated using PyVOL^25^ using default settings with manual curation to encompass the entire ATP-binding pocket. The largest pocket detected in most cases represented the ATP-binding site, to which a 5 Å padding was added for the docking box. DiffDock was executed without restraints on binding site location (i.e., blind docking).

### Ligand Preparation

SMILES strings from the Papyrus data set were transformed into 2D structures using default settings and enantiomers and cis/trans isomers were enumerated using RDKit.^24^ These RDKit objects were converted to.pdbqt format for VinaGPU docking using the Open Drug Discovery Toolkit.^37^ The RDKit objects were written to.csv files in canonical SMILES format with stereo information to use as DiffDock input.

### Docking

Two docking procedures were employed: DiffDock^22^ and AutoDock VinaGPU,^21,39^ both installed through Docker.^23^ Generated VinaGPU poses were converted to mol-format using RDKit.

### AutoDock VinaGPU

A Docker image of AutoDock VinaGPU^21,40^ was used, running on commercial RTX4070 or RTX3070 GPUs. For each protein, the corresponding KLIFS structures with predefined binding site boxes were iterated and all compounds with known activities docked. The AutoDock VinaGPU implementation differs slightly from the well characterized CPU version in its docking settings, where the *exhaustiveness* parameter is now replaced by *search_depth* and *thread*. A small parameter optimization was performed to benchmark the performance of VinaGPU on this data set, resulting in the final settings *search_depth* = 10, *threads* = 8192 which resulted in balanced performance vs run time (data not shown). Output.pdbqt formatted poses were converted to.mol format using OpenBabel and aggregated in a tabular format for inclusion in the database.

### DiffDock

The original DiffDock Github release of October 2021 was used. Compounds were provided in canonical SMILES format with explicit stereochemistry. ESM embeddings were generated using the provided scripts and default settings:
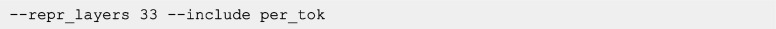


For inference, the release inference.py script was used with minor changes relating to the output data structure. The author recommended settings for high throughput inference were used:
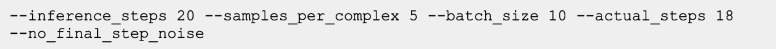


Output.sdf formatted poses were expanded to.mol format and aggregated in a tabular format for inclusion in the database.

### Clash-Score Filtering

The filter criterion (clash <10) was based on the Vina output, where after fitting a normal distribution on the clash scores a 3σ upper limit was calculated to be 10.02, which was visually inspected to be sensible and used for both docking algorithms. The clash-score was calculated per atom using the formula:
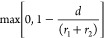
where d is the Euclidian distance between the atoms, and r_1_ and r_2_ are the van der Waals radii of the respective atom types.^41^ This per-atom contribution was calculated based on selections made using the PyMOL API. In brief, KLIFS.pdb and docking pose.mol-files were loaded in PyMOL. A selection around 4 Å of the ligand was made, and for all resulting atoms pairs the clashing contribution was determined. All contributions were summed to yield the pose clash-score.

### Machine Learning

All machine learning algorithms were implemented in PyTorch 2.0. Splits were curated to ensure that the test set pChEMBL distribution is similar to the train set distribution. All DNNs were 3-layer fully connected NNs with the input layer either 2048 bits (ECFP) or 65536 bits (PLEC) to 4000, the hidden layer 4000 inputs to 1000 outputs and the output layer using the 1000 inputs to 1 output value. All layers use ReLU activation functions and the input and hidden layers use a dropout rate of 25% during training. The learning rate is fixed at 10^–5^, batch size 128 with 100 epochs as fixed termination. After every epoch the performance on the test set is evaluated and the best model is stored. Typically, 50–70 epochs are required to reach a plateau.

### Prediction Aggregation

For any given kinase-compound combination, the top 3 poses for all available KLIFS structures were scored by the DNN. To get to a final activity prediction, we tested several aggregation strategies. Taking the mean value of all options (aggregating the various KLIFS, all available stereoisomers, and the top 3 poses) yielded consistently the highest *R*^2^ (Figure S8). As expected, using only the top 1 pose (according to Vina or DiffDock ranking) performed slightly better than taking only the second or third ranked pose, showing that on average the built-in scoring mechanism of both algorithms is able to prioritize the most relevant poses. However, averaging either the top 2 or top 3 poses consistently improved the performance.

## Data Availability

The 3D structure database generated as part of this work is available as an .sqlite database on Zenodo (10.5281/zenodo.10894122), together with the KNIME workflow that provides a simple user interface to search it. Code to reproduce the work described in this paper is available on GitHub (https://github.com/APAJanssen/KinaseDocker2-Paper-code). The PyMOL plugin is available on its own GitHub (https://github.com/APAJanssen/KinaseDocker2), which contains instructions on how to set up the Docker environment. The Docker image is available on Docker Hub (https://hub.docker.com/repository/docker/apajanssen/kinasedocker2).

## Supporting Information Available

Regression statistics for the individual kinases in Tables S1–S3 (XLSX)
Additional materials including Figures S1–S8 (PDF)
