# Supplementary material for: Docking-Informed Machine Learning for Kinome-wide Affinity Prediction
Source: J Chem Inf Model. 2024 Dec 10;64(24):9196–204. doi: 10.1021/acs.jcim.4c01260 (PMC11684025; doi:10.1021/acs.jcim.4c01260)
Supplement: Supplementary file 2 — ci4c01260_si_002.pdf [file ci4c01260_si_002.pdf]

Supplementary Information accompanying

Docking-informed machine learning for kinome-wide affinity prediction

Jordy Schifferstein[1,2], Andrius Bernatavicius[3], Antonius P.A. Janssen*[1,2]
[1] Department of Molecular Physiology, Leiden Institute of Chemistry, Leiden University, The Netherlands
[2] Oncode Institute, The Netherlands
[3] Leiden Institute of Advanced Computer Science, Leiden University, the Netherlands
* E-mail: a.p.a.janssen@lic.leidenuniv.nl

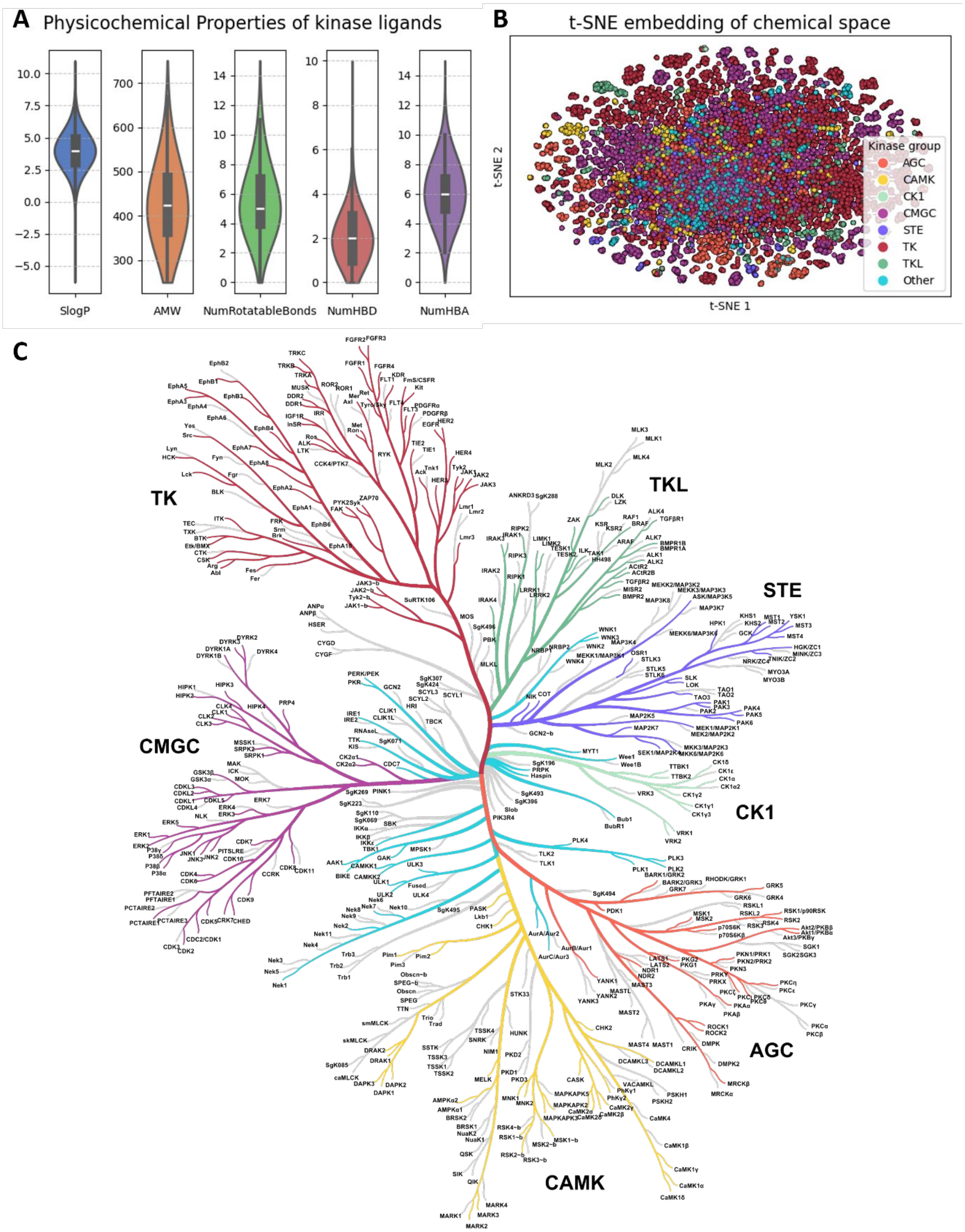

Figure S1: Chemical and kinase diversity | A) Violin plot of physicochemical properties of the kinase inhibitors; B) t-SNE embedding of the chemical space by ECFP4 fingerprints (2048 bits), coloured by majority kinase group target; C) View of included kinases coloured in phylogenetic tree.

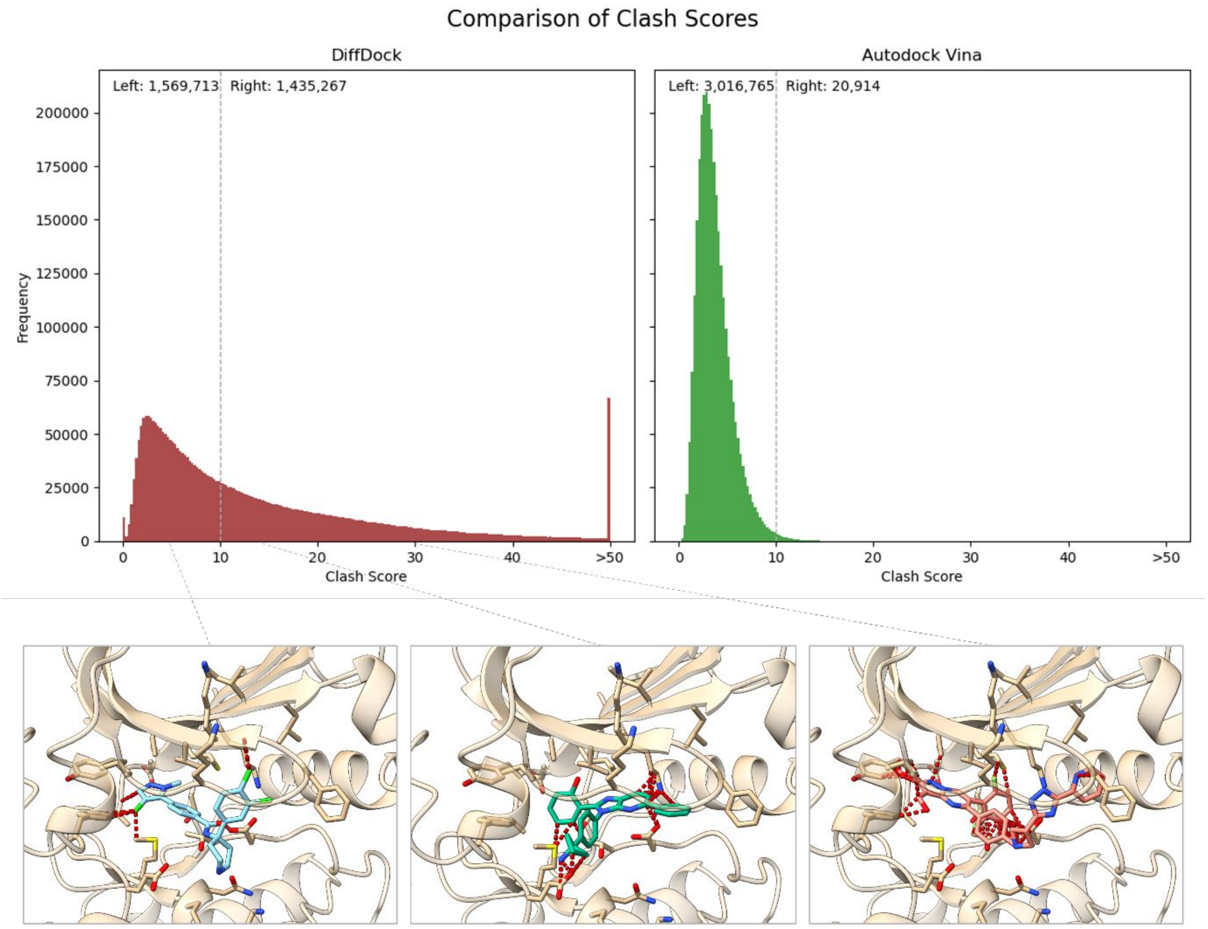

Figure S2: Clashing score filtering | Histograms of the calculated clash scores for all DiffDock poses (left) and VinaGPU poses (right), illustrating the cut-off value of 10. Bottom inset shows 3 illustrations of poses with clash scores of 5, 15 and 30. Red dashed lines indicate atomic clashes. Insets were generated using UCSF ChimeraX[39].

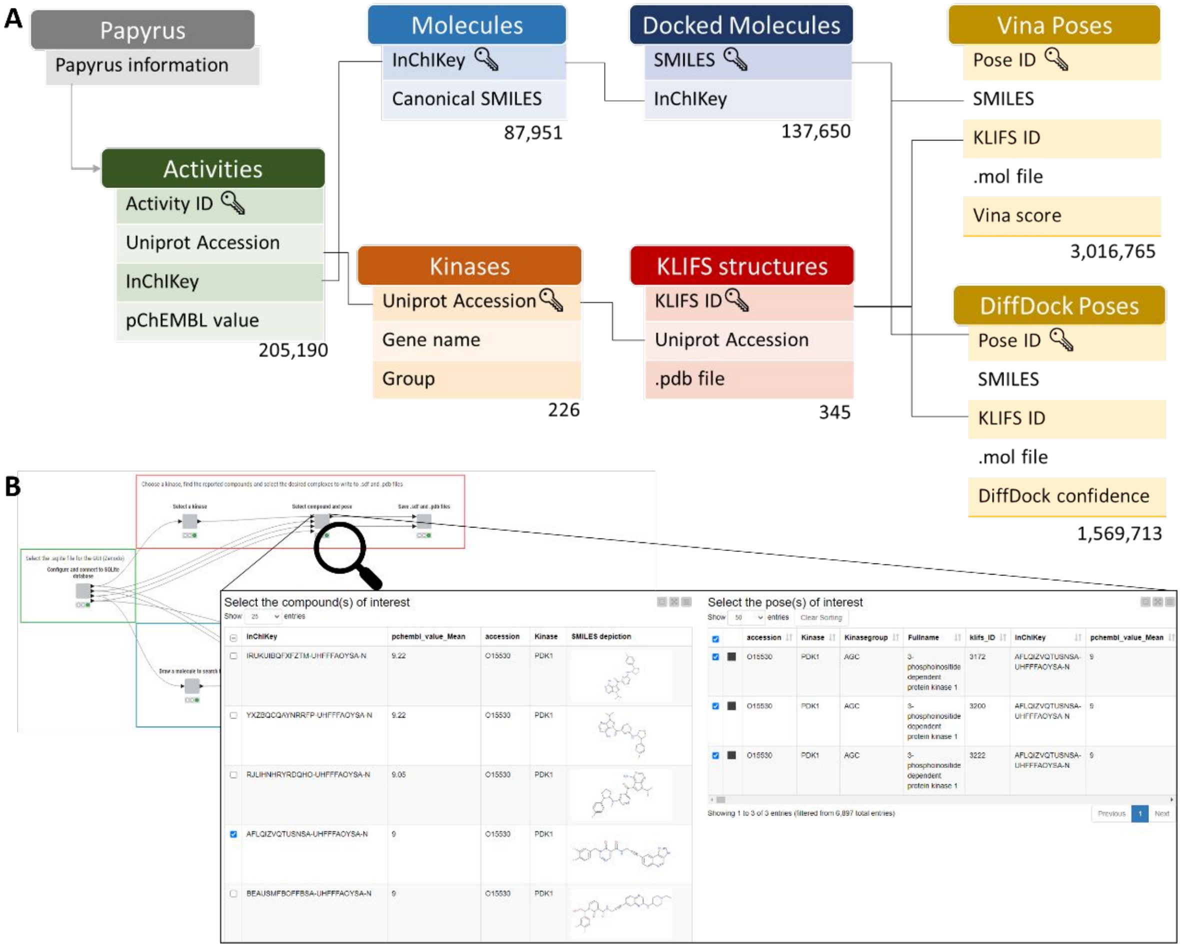

Figure S3 Machine Learning-ready database of kinase-inhibitor complexes | A) Schematic and abbreviated database schema with statistics per table; B) Screenshots of the KNIME-based GUI that enables users to search and download data locally from the database.

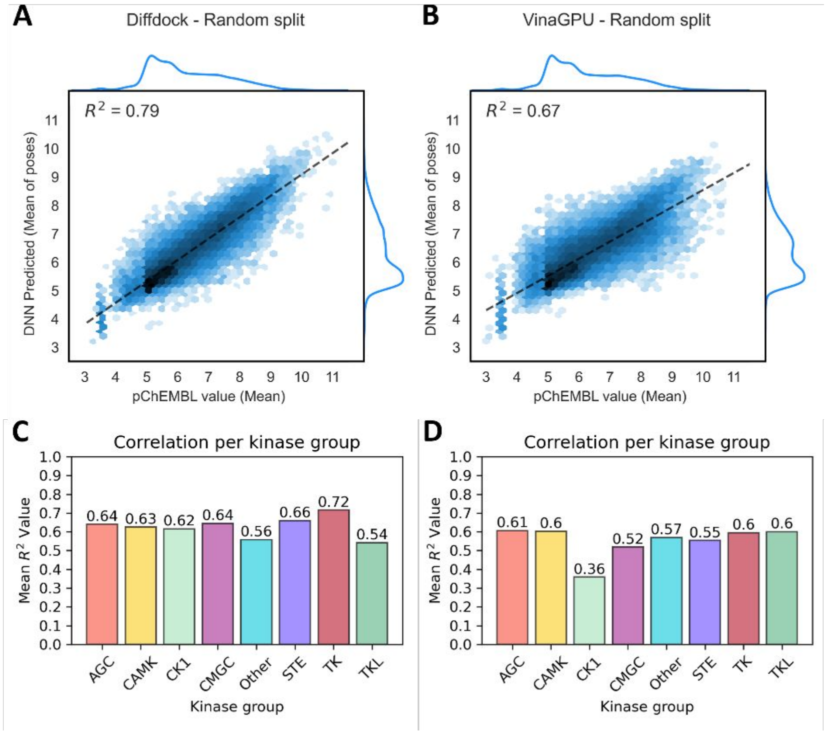

Figure S4: Model performance on the random split | Predicted affinity values vs. literature values for the random-split test set displayed as logarithmic hexbin plots, as based on predictions of the DNN trained on DiffDock poses (A) and on the VinaGPU poses (B). Panels C and D show the average performance per kinase group for DiffDock and VinaGPU models, respectively.

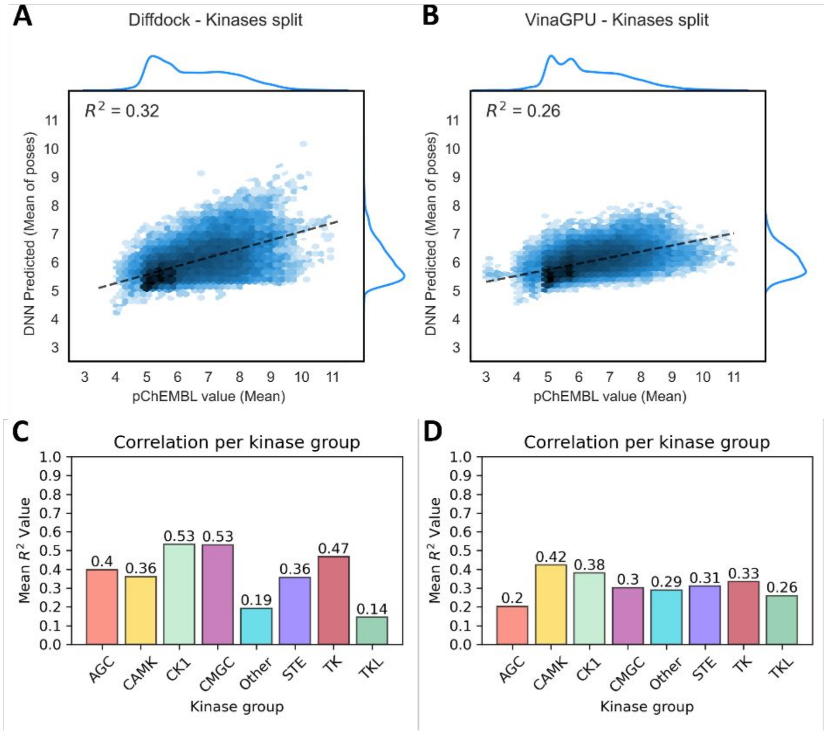

Figure S5: Model performance on the kinases split | Predicted affinity values vs. literature values for the kinase-split test set displayed as logarithmic hexbin plots, as based on predictions of the DNN trained on DiffDock poses (A) and on the VinaGPU poses (B). Panels C and D show the average performance per kinase group for DiffDock and VinaGPU models, respectively.

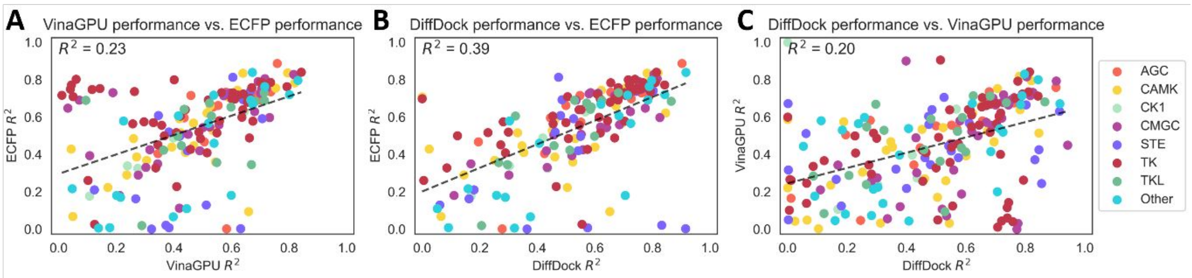

Figure S6: Performance correlation between models | Assessment of the correlation between the per kinase performance for VinaGPU and ECFP (A), DiffDock and ECFP (B) and DiffDock and VinaGPU (C) models. Kinases are coloured by kinase group.

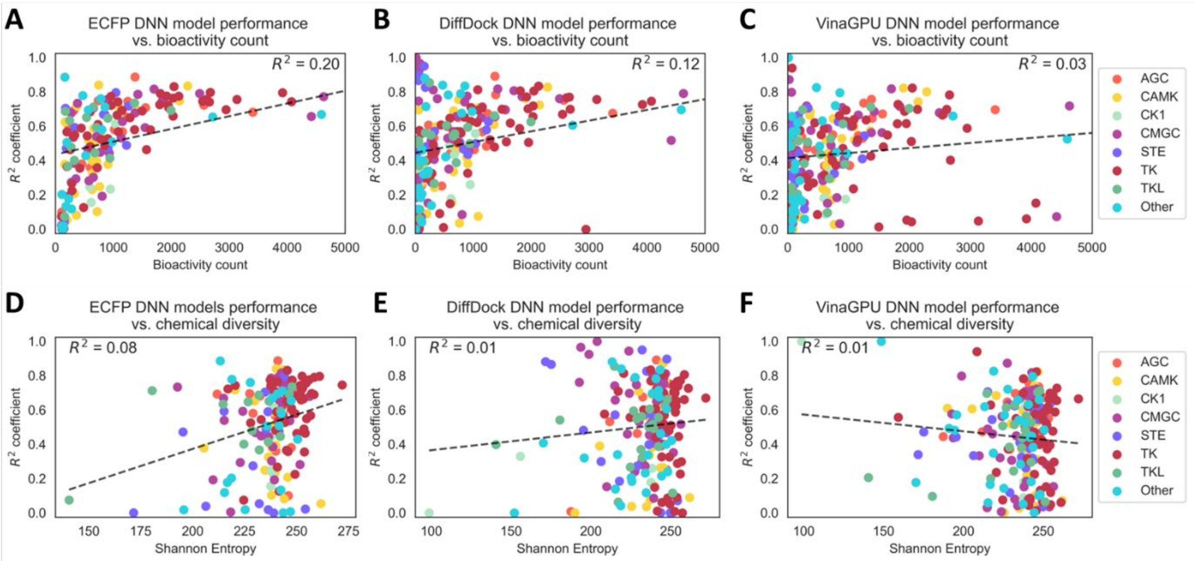

Figure S7: Correlation between performance and bioactivity count or chemical diversity | Assessment of the correlation between the per kinase performance for ECFP and bioactivity count (A), DiffDock and bioactivity count (B) and VinaGPU and bioactivity count (C), ECFP and chemical diversity (D), DiffDock and chemical diversity (E) and VinaGPU and chemical diversity (F). Chemical diversity is calculated as the Shannon entropy (higher = more diverse) of the ECFP fingerprint for all compounds included in the kinase's bioactivity data. Kinases are coloured by kinase group.

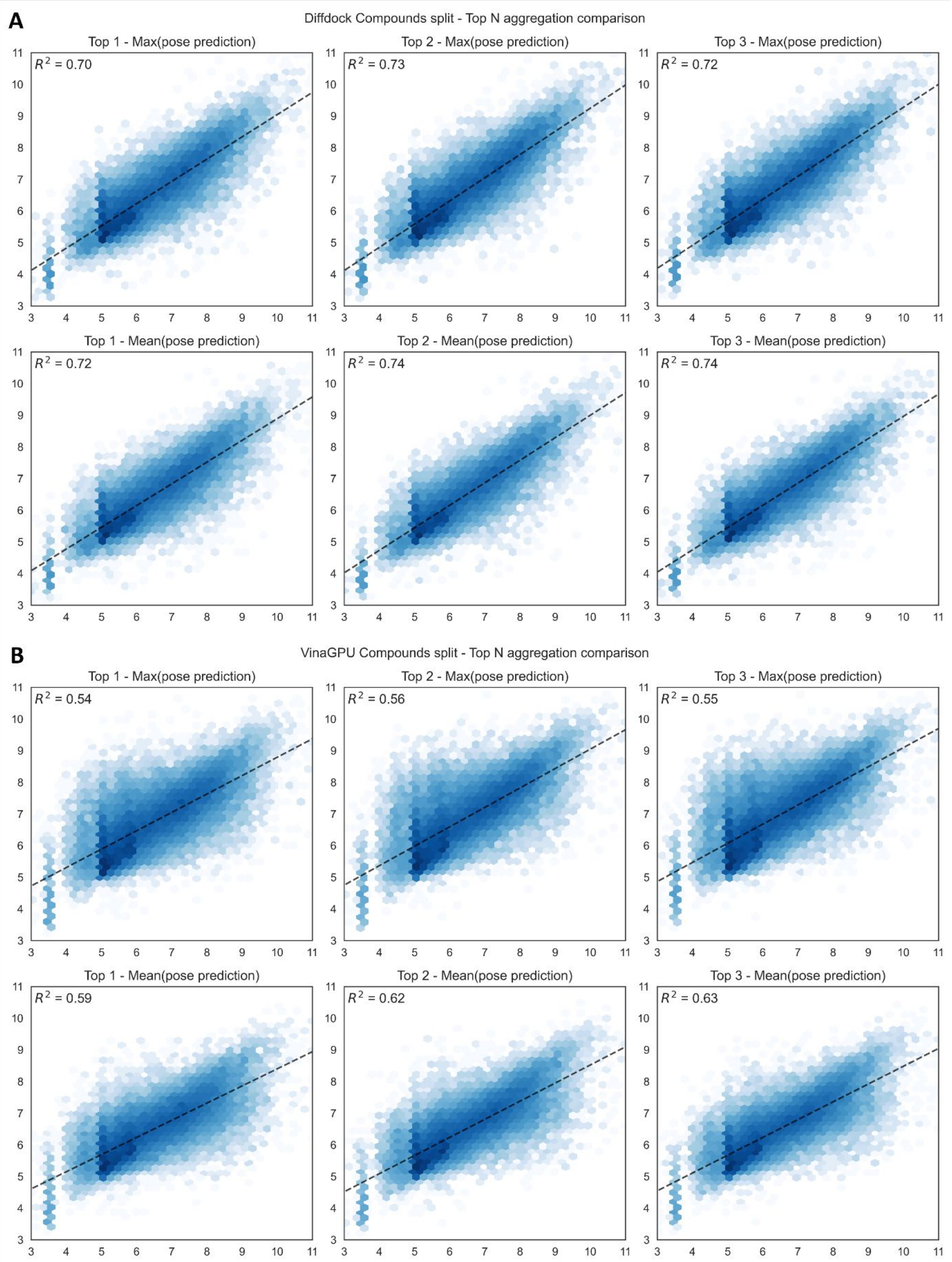

Figure S8: Top N pose aggregation strategies | Predicted affinity values vs. literature values for the compounds-split test set displayed as logarithmic hexbin plots, as based on predictions of the DNN trained on DiffDock poses (A) and on the VinaGPU poses (B). Each sub-plot shows the aggregation of either the Top 1, 2 or 3 poses, using either the maximum or the mean of all predictions for all poses for that compound-kinase pair.
